# Utilization of nanomaterials in MRI contrast agents and their role in therapy guided by imaging

**DOI:** 10.3389/fbioe.2024.1484577

**Published:** 2024-11-19

**Authors:** Wenjia Wang, Shufan Shang, Ye Wang, Bing Xu

**Affiliations:** Department of Radiology, Beijing Shunyi District Hospital, Shunyi Teaching Hospital of Capital Medical University, Beijing, China

**Keywords:** nanomaterials, MRI, contrast agent, therapy, diagnosis

## Abstract

Magnetic Resonance Imaging (MRI) is a globally acknowledged diagnostic procedure particularly recognized for its superior soft tissue contrast, high-resolution imaging, and non-ionizing radiation properties, making it an indispensable tool in the medical field. However, to optimize MRI’s sensitivity and specificity towards certain diseases, use of contrast agents becomes necessary. Recent developments focus on nanomaterial-based MRI contrast agents to improve diagnostic accuracy and image quality. This review highlights advancements in such agents, including metal oxide nanoparticles, carbon-based materials, gold nanoparticles, and quantum dots. It discusses their roles in MRI-guided therapies like targeted drug delivery, hyperthermia, radiation therapy, photodynamic therapy, immunity-boosting therapy, and gene therapy. Insights into the future potential of MRI contrast agents in imaging medicine are also provided.

## Introduction

Magnetic Resonance Imaging (MRI) is a widely utilized, non-invasive clinical imaging technique recognized for its exceptional contrast in soft tissues and high-resolution capabilities (Berger, 2002). Since its inception in the 1980s by pioneers Paul Lauterbur and Peter Mansfield, MRI has significantly advanced medical diagnostics, earning its developers the Nobel Prize in Medicine in 2003 for their contributions to the field (Dreizen, 2004). While traditional MRI demonstrates good inherent contrast between soft tissues, exogenous contrast media are often used to enhance the differentiation between healthy and diseased tissues, aiding radiologists in identifying abnormalities such as tumors (Bruno et al., 2022).

Currently, most commercially available MRI contrast agents are gadolinium-based compounds, including Gadolinium-DTPA and Gadobutrol (Table 1). However, these agents present several limitations: low relaxation efficiency at low doses, potential adverse reactions in patients, particularly nephrogenic systemic fibrosis in those with severe kidney impairment, and non-specific distribution requiring high concentrations for effective imaging. Thus, there is an urgent need for safer, more efficient MRI contrast media that provide better targeting capabilities (Rudnick et al., 2021; Natalin et al., 2010).

**TABLE 1:** Varieties and characteristics of commercial MRI contrast agents.

Common name	Commercial name	Properties	Molecular formula	Advantages	Disadvantages
Gadolinium-DTPA	Magnevist	Paramagnetic	C_14_H_23_GdN_4_O_9_	High relaxivity, widely used	Risk of nephrogenic systemic fibrosis
Gadobenate Dimeglumine	MultiHance	Paramagnetic	C_22_H_34_GdN_4_O_9_	High relaxivity, beneficial for vascular imaging	Risk of allergic reactions
Gadoteridol	ProHance	Paramagnetic	C_17_H_31_GdN_4_O_7_	Lower risk of nephrogenic systemic fibrosis	Lower contrast enhancement
Gadobutrol	Gadovist	Paramagnetic	C_18_H_31_GdN_4_O_9_	High concentration, high relaxivity	Risk of allergic reactions
Gadopentetate Dimeglumine	Dotarem	Paramagnetic	C_28_H_54_GdN_5_O_20_	High relaxivity, lower risk of nephrogenic systemic fibrosis	Risk of allergic reactions

Nanotechnology has emerged as a transformative force in biomedical applications, offering novel solutions through the development of MRI nano-contrast agents (Hsu et al., 2023; Li et al., 2015). Superparamagnetic iron oxide nanoparticles exemplify this advancement, showcasing high magnetic susceptibility and large relaxivity, which can significantly improve image quality. For instance, recent studies have demonstrated that these nanoparticles can enhance tumor visualization and improve diagnostic accuracy compared to conventional gadolinium-based agents (Li et al., 2013). Coating nanomaterials with biocompatible substances can greatly improve the safety and imaging effectiveness of MRI contrast agents (Thangudu et al., 2022).

Moreover, functionalized nanomaterials can facilitate targeted drug delivery and localized therapies, integrating diagnostic and therapeutic functions into a single platform (Veeranarayanan and Maekawa, 2019). This approach not only allows real-time monitoring of treatment responses but also holds promise for innovative techniques like hyperthermia therapy and photodynamic therapy (Zhuang et al., 2022; Luo et al., 2017). For example, research has shown that gold nanoparticles can be used for enhanced imaging and simultaneous photothermal therapy of tumors, illustrating the dual capability of these agents in cancer care (Yang et al., 2022; Vines et al., 2019).

**FIGURE 1:**
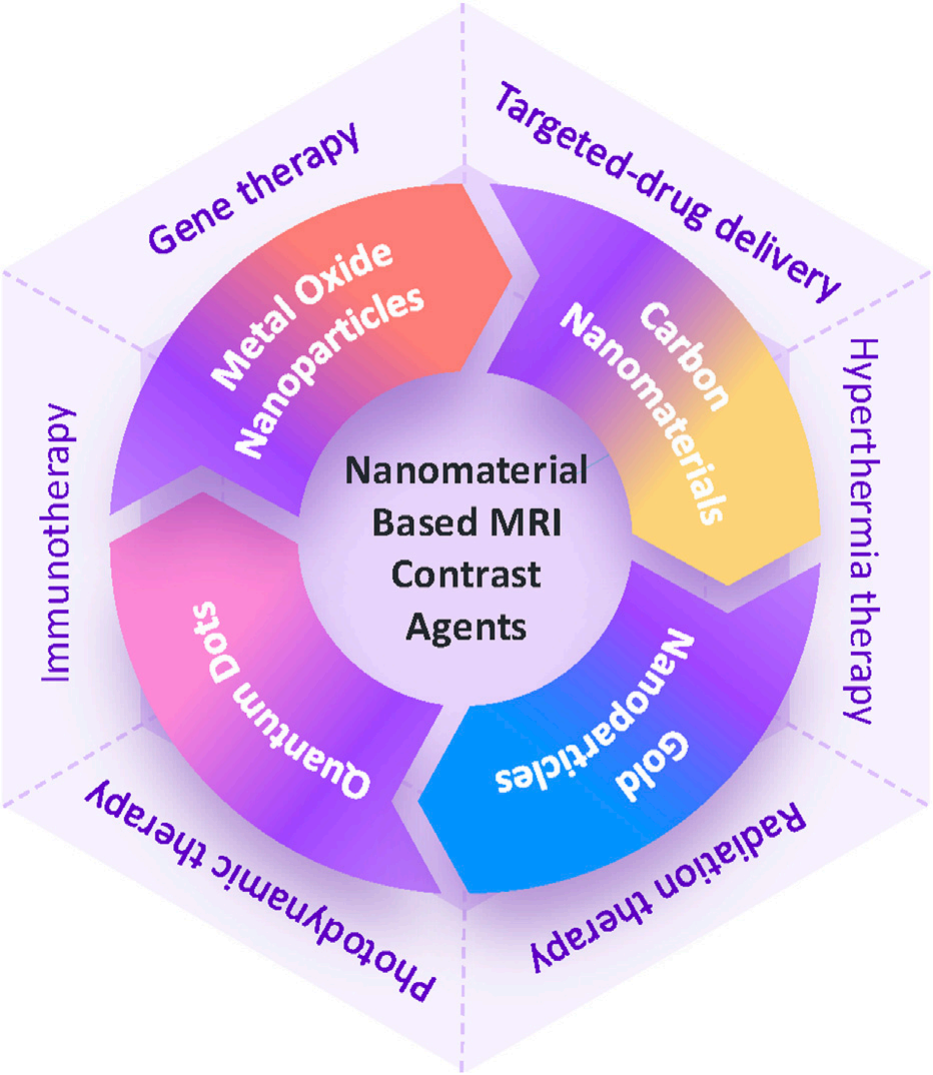
Schematic illustration of MRI contrast agents based on nanomaterials and therapies guided by MRI imaging.

**FIGURE 2:**
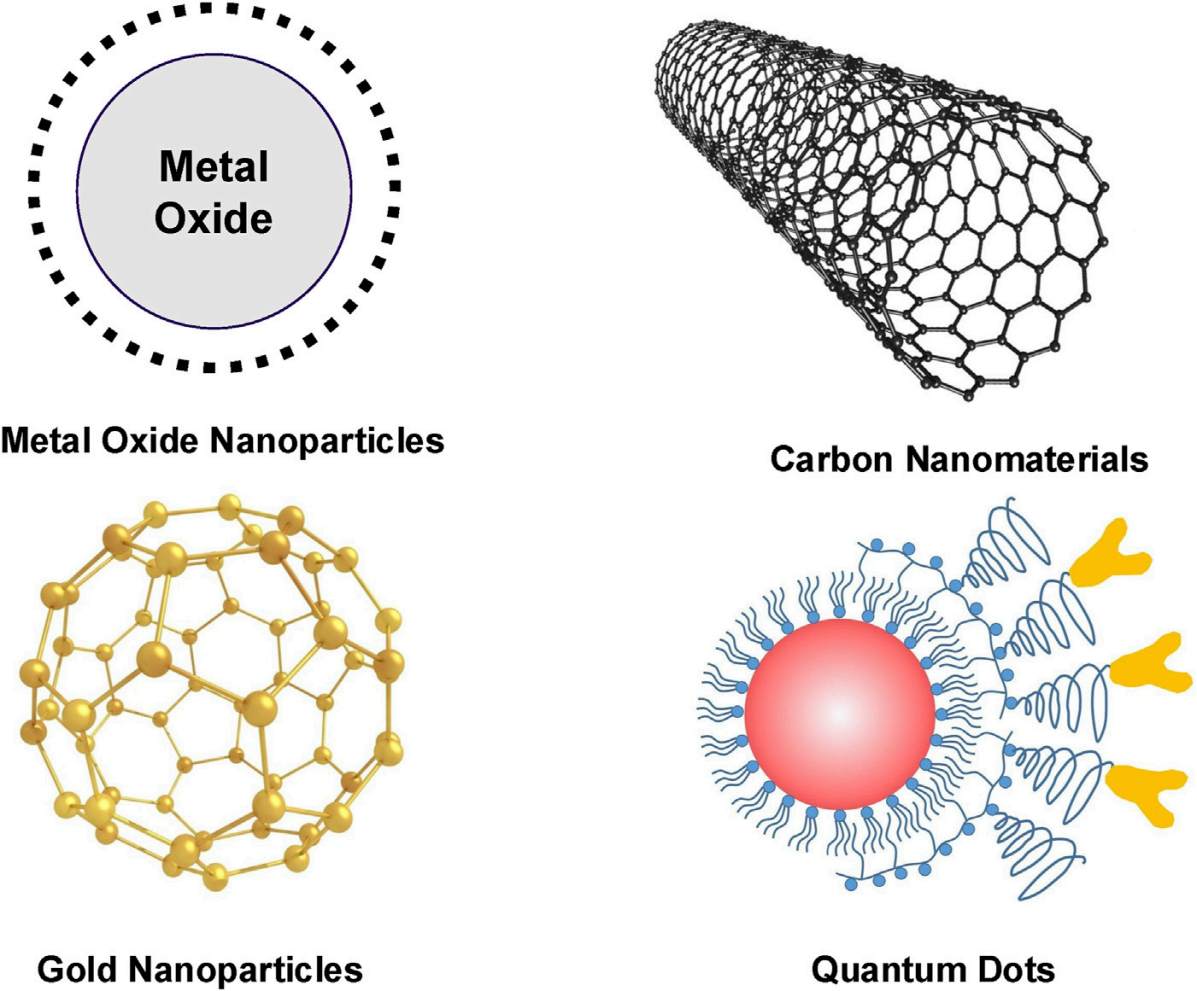
MRI contrast agents derived from the structures of different nanoparticles.

In this article, we explore the evolution of nanomaterial-based MRI contrast agents and their biomedical applications in recent years (Figure 1). We begin by reviewing the current landscape of these agents, focusing on their composition and imaging efficacy (Figure 2; Table 2). We then delve into advancements in MRI image-guided therapies, covering areas such as targeted drug delivery, hyperthermia therapy, radiation therapy, photodynamic therapy, immunotherapy, and gene therapy. Finally, we discuss the challenges and future prospects for nano-based MRI contrast agents in enhancing the effectiveness of medical imaging and therapy.

**TABLE 2:** Comparative table of nanomaterial-based MRI contrast agents.

Type of nanomaterial	Materials	Properties	Size range	Relaxation time (T1/T2)	Applications	Advantages	Limitations
Iron Oxide Nanoparticles	Iron oxide (Fe_3_O_4_, Fe_2_O_3_)	Superparamagnetic	5–100 nm	T2-weighted imaging	Tumor imaging, liver imaging	High relaxivity, minimal toxicity	Limited long-term stability, potential for oxidative stress
Gadolinium-based Nanoparticles	Gadolinium chelates	Paramagnetic	10–300 nm	T1-weighted imaging	Vascular imaging, angiography	High signal enhancement, established clinical use	Nephrotoxicity in patients with renal impairment
Gold Nanoparticles (AuNPs)	Gold (Au)	Biocompatible, non-toxic	1–100 nm	T1 and T2 relaxation effect	Cancer detection, imaging-guided therapy	Enhanced contrast in CT and MRI, versatile applications	Potential aggregation, limited biodistribution
Silica-based Nanoparticles	Silica (SiO_2_)	Stable, tunable surface	50–200 nm	Variable based on dopants	Drug delivery, imaging	Easy modification, good biocompatibility	Low intrinsic relaxivity, requires conjugation with MRI agents
Carbon Nanotubes	Carbon	High surface area, conductivity	1–100 nm	Variable	Targeted drug delivery, imaging	Excellent biocompatibility, multifunctional capabilities	Production challenges, regulatory hurdles
Quantum Dots	Semiconductor materials (CdSe, CdTe)	Size-dependent optical properties	2–10 nm	Variable	Imaging, diagnostics	Bright fluorescence, tunable emission	Cadmium toxicity, limited stability under physiological conditions

## Nanomaterial-based MRI contrast agents

### Metal oxide nanoparticles

#### Superparamagnetic iron oxide nanoparticles

Superparamagnetic iron oxide nanoparticles (SPIONs) are nanoscale particles composed of iron and oxygen, exhibiting magnetic properties that can be controlled and used for various applications such as biomedical imaging (Kandasamy and Maity, 2015). SPIONs are recognized for their T2 contrast attributes, rendering them appropriate for augmenting tissue contrast in MRI (Caspani et al., 2020). Their biocompatibility, stability, and superparamagnetism under magnetic fields have been exhaustively examined, increasing their attractiveness for biomedical implementations (Wei et al., 2021). Moreover, SPIONs have been explored for a range of applications, including targeted drug delivery, cellular engineering, and cell tracking (Singh et al., 2010; Deng et al., 2022). As well, SPIONs have found use as contrast agents in MRI, where their superparamagnetism creates an uneven local magnetic field that leads to shortened T2 values on MRI (Hua et al., 2015). The possible toxicity of SPIONs has also been researched, stressing the need to comprehend their biological impacts (Veiseh et al., 2013).

**FIGURE 3:**
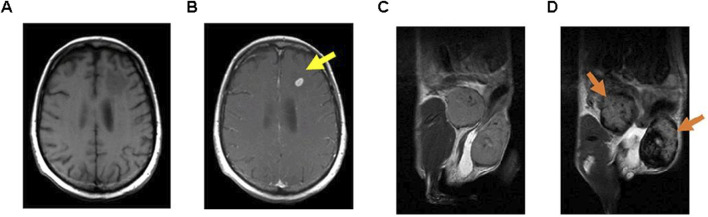
Examples of MRI techniques performed using functionalized GBCA and SPION. T1-weighted MRI images taken **(A)** Before and **(B)** After the administration of GBCA on a brain metastasis in a melanoma patient (15 minutes following injection); T2-weighted MRI images captured **(C)** Prior to and **(D)** After the injection of SPION in inflamed mammary gland tumors of mice (24 hours post-injection). (Reproduced from Serkova (2017), distributed under the terms and conditions of the Creative Commons Attribution [CC BY] license).

In the context of MRI, SPIONs have demonstrated efficacy as contrast agents, notably when engulfed by macrophages in target tissues. They impact the relaxation rate of water, thereby enhancing MRI contrast (LaConte et al., 2007; Daldrup-Link et al., 2011). Additionally, the creation of SPIO-based nanovectors for *in vivo* MRI holds particular interest due to their safe toxicity profile and magnetic characteristics (Veiseh et al., 2013). Furthermore, the employment of SPIONs for targeted diagnosis and therapy in cancers, such as pancreatic cancer, has been explored, emphasizing their potential in medical applications (Figure 3) (Mahmoudi et al., 2011; Demortiere et al., 2011; Serkova, 2017). Various studies have reported the design of SPIO-loaded phospholipid micelles, showcasing the versatility of SPIONs across different delivery systems (Fathy et al., 2019; Cai et al., 2020). Specific findings indicate that PEGylated SPIO nanoparticles exhibit excellent relaxivity and stability, with distinct imaging characteristics depending on the magnetic field strength. For example, 8-nanometer SPIO@PEG5k functions effectively as a T2 contrast agent at 3.0 T and as a dual-mode agent at lower strengths, while 4-nanometer variants are suitable for similar roles at various field strengths (Deng et al., 2021). Additionally, SPIOs with a 14-nanometer core and PEG1000 coating demonstrate a remarkable T2 relaxivity of 385 s^-1 mM^-1, indicating high efficiency per iron atom and significant potential for *in vivo* tumor imaging (Tong et al., 2010). Furthermore, a novel non-gadolinium-based nanoscale contrast agent, SPIO@[Mn (Dopa-EDTA)]2−, has shown promising MRI performance across different magnetic fields, particularly as a dual-modality agent for imaging human organs and blood vessels (Wu et al., 2020).

SPIONs have been extensively researched for their role as contrast agents in MRI (Li et al., 2017). Reports indicate successful incorporation of Doxorubicin along with clusters of SPIONs within micelle cores (Nasongkla et al., 2006). SPIONs are commercially available and have FDA-approval for use in humans (Mailänder et al., 2008). Due to their good biocompatibility and excellent MRI properties, SPIONs are widely utilized in theranostic applications (Yuan et al., 2018). Development efforts have focused on multifunctional, water-soluble SPIONs designed for targeted drug delivery and dual-modality imaging of tumors expressing integrin αvβ3, using PET/MRI techniques (Yang et al., 2011). SPIONs are unique medical nanomaterials with super-magnetic, surface modification, and targeting capabilities. For cell labeling, SPIONs with sizes ranging from 80 to 180 nm are frequently used as T2 and T2*-shortening MRI contrast agents (Skachkov et al., 2018). These multifunctional nanoparticles, referred to as SPIONs-Au, exhibit superparamagnetic properties and have substantial absorbance in the Near-Infrared range of the electromagnetic spectrum (Ji et al., 2007). SPIONs have been widely applied in MRI for imaging blood vessels and tumors, surpassing Gd-based agents (Lu et al., 2014). SPIONs are becoming viable options for nanomedicine focused on tumor imaging and targeted drug delivery for cancer treatment (Zou et al., 2010). With their unique paramagnetic characteristics, they can function as contrast agents across various parametric MRI techniques, including T2 and susceptibility-weighted imaging (Lee et al., 2021). Additionally, SPIO holds great potential for developing smart MRI probes aimed at cell dynamics tracking (Toyota et al., 2012). SPIONs can effectively label neural precursor cells and hematopoietic stem cells (Hu et al., 2009).

#### Ultrasmall superparamagnetic iron oxide nanoparticles

Ultrasmall superparamagnetic iron oxide nanoparticles (USPIONs) have core diameters of less than 5.0 nm and are anticipated to represent a new class of contrast agents. Their promising application stems from their enhanced MRI performance, prolonged circulation time in the bloodstream after suitable surface modifications, ability for renal clearance, and outstanding biosafety profile (Wang et al., 2017; Si et al., 2022). This makes them particularly promising for usage in MRI, a technique known for providing detailed morphological and functional information about atheromatous plaques (Sadat et al., 2017).

These iron oxide agents exhibit signal loss that depends on their concentration in T2-and T2*-weighted MRI sequences, positioning them as potential future contrast agents for patients susceptible to nephrogenic systemic fibrosis (Neuwelt et al., 2009). Macrophages, the predominant inflammatory cells found in stroke lesions, can be effectively detected using USPIONs as a cell-selective MRI agent (Saleh et al., 2004). In research focused on inflammatory responses following ischemic strokes, USPIONs were administered after MRI scans conducted on day 6 (DeLong et al., 2022).

Furthermore, USPIO nanoparticles have sparked interest as potential MRI contrast agents in tumor diagnosis (Wang et al., 2023). Monodisperse USPIONs cores, produced via the thermal decomposition of iron (III)-oleate precursor, have been designed as gadolinium-free T1 contrast agents for vascular imaging (Khandhar et al., 2018). A study examined the effectiveness of R2* and quantitative susceptibility mapping in quantitatively evaluating the accumulation of USPIONs particles in the arcAβ mouse model of cerebral amyloidosis (Klohs et al., 2016). Optimized USPIO contrast agents, designed for uptake by the mononuclear phagocytic system, are gaining increasing attention within translational cardiovascular research (Morris et al., 2008).

#### Iron oxide nanoparticles

Iron oxide nanoparticles (Fe_3_O_4_ NPs) are utilized in MRI as contrast agents, leveraging their magnetic characteristics to enhance imaging quality and enable the visualization of specific tissues or organs with increased sensitivity and specificity. Iron oxide nanoparticles, specifically Fe_3_O_4_, have sparked significant interest due to their potential utility as MRI contrast agents. Study has demonstrated that Fe_3_O_4_ NPs effectively enhance contrast in T2-weighted MRI images (Shou et al., 2020). Moreover, due to their magnetic characteristics and biological compatibility, Fe_3_O_4_ NPs have found extensive use in drug delivery and MRI (Zhang X. et al., 2016).

Explorations into biofunctionalization of Fe_3_O_4_ NPs have opened new avenues for applications in magnetic hyperthermia and MRI (Hayashi et al., 2010). Furthermore, highly-magnetic iron carbide nanoparticles have shown promise as potent T2 contrast agents, suggesting the possibilities for Fe_3_O_4_-based contrast agents in MRI (Huang et al., 2014a). Fe_3_O_4_ mesoporous carbon nanoparticles have been engineered as efficient tumor-targeting nanocarriers for enhancing chemotherapy and photothermal therapy, underscoring their potential in cancer treatment and imaging (Qian et al., 2021). In this context, recent research investigates a novel approach to conjugate modified gefitinib, a primary medication for non-small cell lung cancer, with Fe_3_O_4_ NPs to improve the delivery efficiency of chemotherapy drugs. The study aims to examine how the drug is absorbed in the inner and outer regions of tumors using MRI guidance, with the goal of achieving more effective cancer treatments. This dual application of Fe_3_O_4_ NPs highlights their versatility in both enhancing imaging techniques and facilitating targeted drug delivery, ultimately contributing to improved outcomes in cancer therapy (Thangudu et al., 2023).

Moreover, research has delved into the role of Fe_3_O_4_ mesoporous carbon nanoparticles in MRI enhancement and hyperthermia for cancer treatment (Lu et al., 2021). Investigations have also centered on the optical and magnetic characteristics of superparamagnetic Fe_3_O_4_ mesoporous carbon nanoparticles, illustrating their potential across various applications, including MRI (Xie et al., 2024). Molecular dynamics simulation studies have furnished insights into the adsorption of diverse biopolymers on Fe_3_O_4_ mesoporous carbon nanoparticles, further enriching our understanding of their potential as MRI contrast agents (Zheng et al., 2021). Additionally, magnetic particle imaging has been studied for cell tracking, with superparamagnetic iron oxide nanoparticles, including Fe_3_O_4_ mesoporous carbon nanoparticles, being prevalently used for this purpose (Sehl et al., 2020).

#### Manganese oxide nanoparticles

Manganese oxide nanoparticles (MnO NPs) have garnered significant interest as potential contrast agents for MRI, due to their unique characteristics that position them as apt candidates for enhancing imaging capabilities. Nano-encapsulated manganese oxide (NEMO) particles exhibit properties like pH-switchable signals, high metal loading capacity, and targeting capabilities that render them ideal for specific imaging applications (Martinez de la Torre et al., 2023). Inorganic manganese oxide particles and manganese carbonate have demonstrated versatility in molecular and cellular MRI as convertible contrast agents. Their effectiveness as T1 MRI contrast agents for preclinical research further underscores their potential in diagnostic imaging (Poon et al., 2021).

Manganese-based nanostructures’ responsiveness as MRI contrast agents allows tunable performance for enhanced imaging capabilities in medical diagnostics (García-Hevia et al., 2019). The creation of theranostic liposomes modified with the AS1411 aptamer, which co-encapsulate manganese oxide nano-contrast agents and paclitaxel, presents an innovative approach for cancer imaging and treatment (Li et al., 2019). Studies have demonstrated that folic acid-modified Mn_3_O_4_@SiO_2_ nanoparticles exhibit high colloidal stability and effective targeting of HeLa cells, leading to significant tumor accumulation and enhanced MRI signals (Yang et al., 2014). Similarly, H-MnO_2_-PEG/C&D nanoparticles decompose in acidic environments to release drugs while significantly improving T1 contrast in MRI, showcasing their potential for passive tumor targeting and synergistic therapeutic effects through combined chemotherapy and photodynamic therapy (Yang et al., 2017). Furthermore, FA-Mn_3_O_4_@PDA@PEG nanoparticles exhibit an ultrahigh longitudinal relaxivity of 14.47 mM ^-1s^-1—nearly three times that of commercial agents—demonstrating their efficacy as both MRI contrast agents and therapeutic platforms under near-infrared laser irradiation (Ding et al., 2016). Manganese oxide nanostructures have shown high signal enhancements and responsiveness as MRI contrast media, demonstrating their prospective *in vitro* imaging techniques (Bañobre-López et al., 2018).

Manganese-engineered iron oxide nanoparticles' paramagnetic properties have facilitated the tunability of T1 and T2 contrast abilities, leading to the development of versatile MRI contrast agents with superior imaging capabilities (Huang et al., 2014b). Lipid-micelle-based theranostics loaded with manganese have been studied for their ability to deliver drugs and genes to the lungs simultaneously, highlighting the potential of manganese compounds as multifunctional agents for both imaging and therapeutic uses (Howell et al., 2013). Additionally, research into fruit juices high in manganese ions as oral contrast media for MRI imaging of the gastrointestinal tract illustrates the various sources of manganese-based contrast media (Rizzo et al., 2021).

### Carbon nanomaterials

#### Carbon nanotubes

Carbon nanotubes (CNTs), composed solely of carbon atoms and structured into cylindrical shapes, are renowned for their extraordinary robustness, elevated electrical conductivity, as well as distinct mechanical and thermal attributes (Zhang et al., 2021). In the realm of MRI, CNTs have risen to prominence given their potential to enhance contrast agents, thus improving image precision and allowing superior tissue and organ visualization. These nanotubes encompass single-walled carbon nanotubes (SWCNTs) and multi-walled carbon nanotubes (MWCNTs), both attracting significant research attention (Deshmukh et al., 2020; Tomczyk et al., 2020). To further augment their magnetic properties and suitability for MRI contrast enhancement, strategies such as integrating materials like gadolinium nanoparticles into carbon nanotubes have been undertaken (Gizzatov et al., 2015). MWCNTs, with their distinguished magnetic and electronic traits, morphology, and capability to breach cell membranes, stand out as attractive candidates for MRI contrast agents (Maciejewska et al., 2015).

Research indicates that the encapsulation of gadolinium ions within carbon nanotubes can amplify MRI contrast for cellular imaging. This highlights the potential role of these nanotubes in propelling further advancements in imaging technologies (Marangon et al., 2014). Additionally, modifying carbon nanotubes with specific ligands, such as hyaluronic acid, could facilitate targeted delivery of contrast agents to tumorous cells, thereby enhancing MRI’s diagnostic abilities (Hou et al., 2015). The synthesis of carbon nanotubes with other materials like iron oxide nanoparticles brought about the creation of dual-functional contrast media suitable for MRI and alternate imaging modalities, thus accentuating the adaptability of carbon nanotubes in a range of multimodal imaging applications (Wang et al., 2014).

#### Fullerenes

Fullerenes are carbon-based molecules with a cage-like structure, typically formed in spherical or tubular shapes. They exhibit distinct characteristics, including remarkable stability and strong antioxidant properties (Minami et al., 2021). For instance, one study highlights the use of water-soluble endohedral fullerenes as MRI contrast media, showcasing intriguing aspects of this research (Dunsch and Yang, 2007). In addition, work by Ghiassi et al. discusses the use of gadolinium-loaded endohedral fullerenes as effective relaxation agents for MRI, further emphasizing their potential for biomedical imaging applications (Ghiassi et al., 2014). Moreover, research by Shu et al. presents a straightforward preparation of a novel gadofullerene-based MRI contrast media with high relaxivity, spotlighting the potential of fullerenes for enhancing MRI contrast (Shu et al., 2009). The studies also offer insights into the preparation, functional modifications, and life science applications of fullerenes, particularly in context of MRI contrast enhancement. The potential of fullerenes for tumor imaging, drug delivery, and antioxidant capability has also been discussed, further accentuating their multifunctional capabilities and potential for biomedical applications (Gaur et al., 2021; Alosaimi et al., 2023). Furthermore, these studies address the challenges and opportunities related to using fullerenes as MRI contrast media, emphasizing their unique physical characteristics and potential technological uses in medical imaging (Bolskar et al., 2003).

#### Graphene

Graphene consists of a single layer of carbon atoms organized in a two-dimensional honeycomb lattice, recognized for its remarkable strength, electrical conductivity, and transparency. Hybrid nanomaterials based on graphene have been utilized in various diagnostic imaging techniques, including fluorescent imaging, MRI, CT scans, and radionuclide imaging (Akinwande et al., 2017). Hybrid nanoparticles of Mn_3_O_4_–PDA–graphene quantum dot have demonstrated potential for T1-weighted MRI and optical imaging-assisted photodynamic therapy (Cheng et al., 2019). Earlier research has suggested that Mn^2+^ carbon nanostructure complexes made from graphene oxide nanoplatelets can function effectively as MRI contrast media (Xiao et al., 2016). The superior transmittance and photoluminescence behaviors make graphene a highly appealing nanomaterial for applications in MRI and biomedical imaging (Cheng et al., 2019). Graphene’s potential for diverse, electromagnetic-transparent electronics in medical imaging and diagnostics goes beyond that of conventional metal electrodes (Tweedie et al., 2020). Moreover, by incorporating specific polar C─F bonds into the small-sized graphene oxide framework, a highly water-soluble nanoprobe with exceptional MRI capabilities has been created, providing high-resolution anatomical information in MRI (Xu et al., 2024). Coating copper microelectrodes with graphene has been shown to reduce toxicity, allowing for the development of MRI-compatible neuroelectrodes (Fairfield, 2018).

#### Silicon nanoparticles

Silicon nanoparticles are tiny particles made of silicon, recognized for their distinct optical and electronic characteristics, which render them ideal for various applications, including use as contrast agents and in biomedicine. Studies have indicated their effectiveness in enhancing near-infrared (NIR) imaging in ophthalmology (Ki et al., 2024). The development of hyperpolarized silicon nanoparticles has been recognized as a significant advancement for MRI-based biomedical imaging (Hu et al., 2018). Moreover, the utilization of fluorinated silicon nanoparticles has exhibited promise in cancer detection through 19F MRI and fluorescence imaging (Li et al., 2018). The distinctive characteristics of silicon nanoparticles, such as long T1 relaxation times, have facilitated *in vivo* hyperpolarized silicon MRI with extended imaging durations (Zhang Y. et al., 2016). Additionally, the hyperpolarization of silicon particles using dynamic nuclear polarization (DNP) has notably increased their MRI signals, rendering them effective contrast agents for *in vivo* MRI (Lin et al., 2021). Silicon nanoparticles have been investigated for their promise as background-free MRI contrast agents, owing to their biological compatibility and adaptable surface chemistry (Seo et al., 2018). Despite the progress made, challenges persist in the development of silicon nanoparticles for MRI applications, including issues related to morphology, size regulation, dispersal, and surface alterations (Luu et al., 2022). Nevertheless, the extended spin-lattice relaxation times displayed by hyperpolarized silicon particles make them intriguing candidates for innovative MRI probes (Kwiatkowski et al., 2017).

### Gold nanoparticles

Gold nanoparticles (AuNPs) have garnered considerable attention in cancer diagnosis and treatment because of their unique properties (Vankayala and Hwang, 2018). They are stable, nonimmunogenic, and exhibit low toxicity *in vivo* (Ehlerding et al., 2016). Through passive targeting (enhanced permeability and retention effect) or active targeting, AuNPs can preferentially accumulate in tumors, enhancing imaging sensitivity and therapeutic efficacy. Additionally, PEG or zwitterion-stabilized AuNPs exhibit remarkable blood circulation, resulting in a longer half-life and therapeutic window compared to smaller molecular agents (Huang et al., 2010).

The synthesis and adjustment of the physicochemical properties of AuNPs are quite simple. Based on their size and crystal structure, AuNPs can be categorized as clusters with nanometer-scale diameters or colloidal particles that vary from a few nanometers to several hundred nanometers. Numerous studies have investigated the application of AuNPs as MRI contrast media, with the objective of improving image quality and providing multifunctional platforms for biomedical utilization. The potential of FePt nanoparticles for dual-modal CT and MRI molecular imaging was showcased, underscoring the application of nanotechnology in biodistribution and biocompatibility research (Chou et al., 2010). AuNPs were functionalized as contrast media for both *in vivo* X-ray and MRI scans, accentuating their versatility in providing contrast across various imaging modalities (Alric et al., 2008).

Research has demonstrated the *in vivo* radiosensitizing effects of gadolinium chelate-conjugated AuNPs, highlighting their potential to improve MRI contrast (Miladi et al., 2014). Additionally, hybrid gold-iron oxide nanoparticles were synthesized for use as an advanced platform for MRI contrast agents, exploiting the unique characteristics of each material to optimize imaging (Hoskins et al., 2012). The influence of PEGylation on the cellular uptake and *in vivo* distribution of AuNPs MRI contrast media was examined, suggesting their potential in targeted imaging and biodistribution research using gold nanoparticles (El-Baz et al., 2022). Gd-chelate functionalized AuNPs were designed as contrast media for *in vivo* CT and T1-weighted MRI, showcasing the versatility of AuNPs in multimodal imaging applications (Zhu et al., 2013). Relaxometric studies on variously shaped AuNPs conjugated with Gd-chelates revealed high relaxivity for PEG-Gd(III) gold nanoconjugate platforms, suggesting their efficacy as MRI contrast media (Fatehbasharzad et al., 2020). The usage of hydrogenated ligand-protected AuNPs coated with Gd(III) chelates as 1H-MRI contrast media was reported, highlighting the potential of gold nanoparticles in providing contrast for MRI applications (Şologan et al., 2019). A comparison of AuNPs with iodinated contrast agents demonstrated that AuNPs provide superior contrast enhancement and reduce radiation doses in computed tomography (Taghavi et al., 2020).

### Quantum dots

#### Carbon quantum dots

Carbon quantum dots (CQDs) are nanoscale carbon particles known for their unique optical properties, which make them applicable in numerous fields, including biology, medicine, and electronics. CQDs have attracted significant attention as potential MRI contrast media due to their distinctive characteristics. The role of Gd-doped CQDs as positive MRI contrast media has been underscored, revealing their potential in enhancing imaging sites (Atabaev, 2018). Furthermore, there’s an inference that CQDs are less prone to stimulate bacterial resistance than antibiotics, positing them as appealing options for extensive clinical application (Wu et al., 2021).

Expanding beyond imaging, the potential of CQDs in visible-light-activated bactericidal functions has been examined, signifying their broad-ranging applications (Meziani et al., 2016). Moreover, owing to their excellent conductivity, small size, and ability to accommodate various functional groups, CQDs have emerged as viable electroactive nanomaterials, positioning them as promising candidates for supercapacitor applications (Prasath et al., 2018). However, careful scrutiny is required towards the cytotoxicity of CQDs and their implications for cell imaging if they are to be utilized as cell imaging agents (Wang et al., 2011).

CQDs, particularly those smaller than 10 nm, are considered promising for microbial imaging, detection, and inactivation due to their outstanding optical properties, customizable surfaces, and good biocompatibility (Lin et al., 2019). Additionally, the synthesis of Gd-doped CQDs presents potential as multi-purpose imaging probes and MRI contrast media for clinical assessment and neuroimaging (Molaei, 2022). Additionally, the production of CQDs via hydrothermal treatment is proposed as a novel strategy to boost diaCEST contrast efficiency, thereby broadening the scope of CQDs in MRI (Pandey et al., 2022). The hydrophilicity of C-dots and the abundance of exchangeable protons on their surface have been noted to transform them into effective diaCEST MRI contrast agents, underlining their adaptability in multimodal imaging (Zhang et al., 2019).

#### Gadolinium-doped quantum dots

Gadolinium-doped quantum dots (Gd-QDs) are nanoscale semiconductor particles doped with gadolinium, which exhibit enhanced magnetic properties and can be used for imaging and diagnostic applications. Research on gadolinium-based nanoparticles, such as Gd_2_O_3_, GdF_3_, and GdPO_4_, has demonstrated their potential effectiveness as contrast agents for MRI (Na et al., 2009). Furthermore, the development of gadolinium-based contrast agents linked to specific ligands, such as aptamers and peptides, provides promising opportunities for improved detection of biomarkers like amyloid-β oligomers, which are important in Alzheimer’s disease (Kim et al., 2023; Yu et al., 2021). Additionally, the synthesis of water-dispersible Gd_2_O_3_/graphene oxide nanocomposites has indicated an increase in MRI T1 relaxivity, spotlighting the potential for innovative contrast agent designs (Wang et al., 2015). The search for better MRI contrast media has prompted studies on macromolecular ligands, which show significantly higher relaxivity than standard commercial Gd^3+^ MRI agents (Li et al., 2012). Moreover, research is focused on creating biodegradable macromolecular MRI contrast media to facilitate the excretion of Gd(III) chelates after MRI scans, addressing worries about gadolinium retention in the body (Wu et al., 2009). Investigations into polymer micelles embellished with gadolinium complexes aim to enhance MRI sensitivity for advanced imaging applications (Grogna et al., 2010). Despite the common use of Gd-based contrast media for their T1 enhancement properties, significant concerns regarding potential toxicity and gadolinium retention in the body persist. Studies have detected gadolinium accumulation in the brains and kidneys of individuals who have received these MRI contrast media, highlighting the need for safer and more biocompatible alternatives (DeAguero et al., 2023).

#### Manganese-doped quantum dots

Manganese-doped quantum dots (Mn-QDs) are nanoscale semiconductor particles doped with manganese ions, which impart unique optical and magnetic properties for applications such as imaging and sensing. The distinctive properties of these Mn-QDs, including their fluorescent and magnetic traits, render them appealing for dual-mode imaging applications (Zhou et al., 2018). Particularly, Mn-doped ZnSe QDs have been investigated for their potential in fluorescence/MRI dual-mode imaging, underscoring their suitability for bio-imaging applications (Zhou et al., 2018). Moreover, the ability of Mn-QDs to provide contrast enhancement for both T1-and T2-weighted MRI positions them as versatile candidates for multimodal imaging (Kunuku et al., 2023). Studies on the synthesis of highly luminescent surface Mn^2+^-doped CdTe QDs indicate their potential as multimodal contrast agents that can be used for both fluorescence imaging and MRI (Zhang et al., 2014). Further bolstering the potential of Mn-doped QDs as contrast agents is their magnetic behavior, which makes them apt for light-handled spin-based operations (Echeverría-Arrondo et al., 2009). Additionally, the sensitized chemiluminescence reaction between hydrogen peroxide and periodate of various types of Mn-doped ZnS QDs underscores their potential for diverse bioimaging applications. The multifunctionality of Mn-QDs, offering both fluorescence and magnetic resonance properties, heralds them as promising candidates for advanced imaging modalities (Gharghani et al., 2021).

## MRI image-guided therapy

While modern therapeutic options for cancer prominently feature chemotherapy and radiotherapy, their non-specific nature can lead to substantial collateral damage. The inherent lack of selectivity in chemotherapeutic drugs results in severe harm to normal cells along with the targeted cancer cells. Similarly, radiation employed in radiotherapy can impact nearby organs due to the generation of oxygen-free radicals during the irradiation process. To tackle these concerns, progress in molecular imaging and nanotechnology has resulted in the creation of innovative nanoagents that possess both diagnostic and therapeutic capabilities. These nanoagents are being applied in an array of innovative therapies including targeted drug delivery, hyperthermia therapy, radiation therapy, photodynamic therapy, immunotherapy, and gene therapy, amongst others. These emerging modalities hold the promise of minimizing the side effects associated with traditional therapies while improving treatment efficiency, representing a major advancement in cancer management. In this context, we will concentrate on MRI image-guided cancer therapy. MRI image-guided therapy offers precise visualization and localization of tumors, enabling targeted and minimally invasive treatments. It enables real-time monitoring, permitting prompt modifications during procedures to enhance therapeutic effectiveness while reducing harm to adjacent healthy tissues. Recent advancements in nanoagents have created new possibilities for MRI-guided interventions, setting the stage for more personalized and effective treatment strategies in cancer care.

### Targeted-drug delivery

Targeted drug delivery involves utilizing specialized medications or delivery systems designed to specifically focus on particular cells or tissues within the body, thereby enhancing therapeutic effectiveness (Liang et al., 2021). The application of MRI in targeted drug delivery presents a promising strategy to deliver therapeutics to specific sites, such as tumors, thereby minimizing systemic side effects. Nanoparticles have emerged as essential components in targeted drug delivery systems due to their numerous advantages, including improved drug solubility, enhanced bioavailability, and precise targeting of affected tissues (Wathoni et al., 2020; Yu et al., 2011). These nanoparticles can be designed to carry drugs and guided to the intended site using external stimuli like magnetic fields or by attaching ligands that bind to specific receptors on target cells (Liu et al., 2022; Clark et al., 2016). The adoption of nanoparticles in targeted drug delivery holds potential to overcome hurdles faced by conventional drug delivery systems, such as poor solubility and non-specific toxicity (Jana et al., 2013; Díez-Villares et al., 2021).

The evolution of multifunctional nanoparticles—possessing traits such as pH-responsiveness, superparamagnetism, and fluorescence—has unveiled new opportunities for targeted drug delivery and imaging (Zhou et al., 2011). Such advancements have set the stage for designing nanoparticles capable of not only delivering drugs to specified sites but also enabling real-time monitoring of their distribution within the body. In the realm of MRI-guided targeted drug delivery, magnetic nanoparticles used as drug carriers have garnered significant interest (Patel et al., 2018). These nanoparticles can be directed to the target site using external magnetic fields, enabling precise localization of drug administration. Moreover, combining MRI with targeted drug delivery systems provides the opportunity for non-invasive real-time monitoring of drug distribution and accumulation, offering valuable insights into the effectiveness of the delivery system (Liu et al., 2023). The development of nanoscale drug delivery systems has also paved the way for exploring innovative approaches, such as photocontrolled drug delivery. In this method, nanoparticles are designed to discharge drugs in response to specific light wavelengths, enabling spatiotemporal control over drug release (Jana et al., 2013). This strategy shows promise for achieving precise control over delivery of medication to the target area, further enhancing the specificity and efficacy of the treatment (Combes et al., 2021).

### Hyperthermia therapy

Hyperthermia therapy utilizing MRI involves the use of magnetic nanoparticles to create localized heating, particularly for targeted cancer therapies. Activating these nanoparticles with an external alternating magnetic field has emerged as a promising method for enhancing targeted cancer treatment (Fatima et al., 2021). Magnetic Nanoparticle Hyperthermia (mNPH) holds the advantage of superior tumor targeting over traditional hyperthermia delivery techniques and offers possibilities for better therapeutic ratio (Petryk et al., 2013). The research and development in magnetic nanoparticles for hyperthermia therapy are gathering attention due to their potential role as a thermal therapy in clinical trials treating cancers and other diseases, and in thermally activated drug delivery under an alternating magnetic field (Iglesias et al., 2021). Magnetic hyperthermia represents a non-invasive cancer treatment that leverages the power of magnetic fields to eliminate cancer cells, earning recognition as a useful therapeutic modality in treating malignant tumors (Gao et al., 2010). The use of an external alternating magnetic field to activate magnetic nanoparticles for hyperthermia therapy offers an intriguing method in targeted cancer therapy (Rejinold et al., 2022). On top of this, magnetic fluid hyperthermia, a thermal therapy combining nanotechnology and hyperthermia, appears promising as a heating modality, promoting a therapeutic ratio by confining heating to specifically targeted tissues (Soetaert et al., 2020).

### Radiation therapy

Radiation therapy (RT) involves the application of high-energy radiation to specifically target and destroy cancer cells or reduce the size of tumors. Recently, there has been a significant movement towards integrating MRI to enhance the precision and efficacy of RT. MRI-guided RT systems have proven instrumental, contributing unique imaging benefits that enhance precision in treatment planning (Hu et al., 2020). The exceptional soft-tissue contrast offered by MRI is crucial for accurately defining the gross tumor volume and organs at risk (OAR) during the planning of RT, resulting in improved clinical outcomes for various types of cancer (Metcalfe et al., 2013). Moreover, MRI shows promise in assessing treatment effects, as it can identify late radiation injuries, including left atrial fibrosis, through late gadolinium enhancement imaging (Huang Y. J. et al., 2016).

The incorporation of MRI in RT planning has yielded promising results in an array of cancers, including uterine cervical neoplasms, brain neoplasms, lung neoplasms, and prostate neoplasms (Hu et al., 2020). Specifically, multiparametric MRI, which includes diffusion-weighted MRI, has shown remarkable accuracy in detecting recurrent cancer post- RT, thereby providing invaluable insights for treatment evaluation (Bains et al., 2012). Furthermore, dynamic contrast-enhanced MRI holds promise for initial evaluation of tumor response to RT, thus enabling adaptive treatment optimization based on functional changes (Malamas et al., 2016). The incorporation of MRI into RT processes has propelled the creation of MRI-guided treatment systems, which enable real-time imaging during radiation delivery, allowing high doses of radiation to be targeted at tumors while protecting adjacent organs at risk (Zhong et al., 2023). In addition, MRI-based techniques have been utilized to monitor respiratory-induced organ motion, contributing to the accurate delivery of RT (Lee et al., 2016). However, the application of MRI in RT planning does not come without its challenges. Despite MRI offering superior tissue contrast and functional imaging capabilities, there are inherent limitations and drawbacks to its use in treatment planning, thereby necessitating a mindful approach and thorough evaluation (Heerkens et al., 2017).

### Photodynamic therapy

Photodynamic therapy (PDT) utilizes photosensitizing agents activated by light to generate reactive oxygen species (ROS) that induce cellular damage and apoptosis, demonstrating effectiveness against locally advanced cancerous tumors and actinic keratosis (Cheng et al., 2015; Shafirstein et al., 2018). Recent research highlights PDT’s potential in prostate cancer treatment, alongside the integration of medical imaging techniques like MRI to enhance therapeutic monitoring. This includes developing photosensitizer conjugates that serve dual roles as PDT agents and contrast agents for imaging, thereby aiding in assessing therapy effectiveness (Eisen et al., 2021). Concurrently, studies on SIDP nanomaterials (NMs), which exhibit robust photothermal/photodynamic properties, have shown their efficacy in killing B16-F10 cells *in vitro* and their capability to non-invasively monitor distribution in tumor tissues through MRI. When combined with PD-1 inhibitors, SIDP NMs demonstrate significant promise in treating both primary and metastatic tumors (Wu et al., 2024). Additionally, researchers successfully synthesized a nanoscale photothermal therapeutic agent based on dopamine-modified polyaspartic acid and SPIO, known as SPIO@PAsp-DAFe/PEG nanocomposites. These nanocomposites possess MRI enhancement capabilities, appropriate particle size, higher T2 relaxivity, and near-infrared photothermal conversion efficiency, while maintaining good stability and reproducibility. The distribution of these nanocomposites in tumor areas can be effectively monitored using MRI, facilitating precise treatment protocols for photothermal therapy (PTT). Importantly, SPIO@PAsp-DAFe/PEG inhibited tumor growth in mice without significant toxicity, making it an effective T2-weighted MRI/PTT therapeutic agent (Du et al., 2023). The development of multifunctional nano-micelles for MRI-guided therapies further supports this approach, indicating strong contrast effects that enhance precision in photothermal therapy. Photosensitizer conjugates have been engineered to function both as PDT agents and medical imaging contrast agents. This dual functionality allows visualization before, during, and post-PDT, thereby facilitating the assessment of therapy effectiveness (Celli et al., 2010). Moreover, exploration is underway regarding the optimal MRI methodology for evaluating PDT-triggered histopathological responses in the prostate. These studies suggest that MRI could serve as a valuable tool for monitoring PDT effects (Kulik et al., 2014). Despite these advances, researchers acknowledge limitations in current strategies and recommend ongoing optimization of nano-carrier designs and exploration of combinations with other immunotherapies to improve outcomes (Zeng et al., 2013).

### Immunotherapy

Immunotherapy refers to the use of the immune system to treat diseases, typically by stimulating or enhancing the body’s natural defense mechanisms against infections or cancer (Hegde and Chen, 2020). MRI-guided therapy has become an integral part of modern cancer immunotherapy, offering a multifaceted approach to enhance the precision and effectiveness of treatments. The high-resolution soft tissue imaging capabilities of MRI allow for the accurate localization of tumors and the monitoring of therapeutic responses, which is crucial for the gradual process of immune system activation against cancer cells (Kang et al., 2023). This precision is further enhanced by the ability to guide minimally invasive procedures, ensuring the precise delivery of immunotherapeutic agents directly to the tumor site and reducing systemic side effects (Nishino et al., 2019).

The combination of MRI-guided therapies with immunomodulatory agents, such as immune checkpoint inhibitors, enables real-time monitoring of the effects on tumor burden and immune cell infiltration. This allows for the adjustment of treatment strategies, optimizing the potential for an effective immune response (Wei et al., 2019). Moreover, certain MRI-guided therapies can induce immunogenic cell death, promoting the exposure of tumor antigens and the release of damage-associated molecular patterns (DAMPs), which stimulate an antitumor immune response (Fu et al., 2022).

Personalized treatment planning is significantly improved with MRI, as the detailed imaging helps in understanding the tumor’s microenvironment and its interaction with the immune system. This understanding allows for the tailoring of treatments to maximize the potential for an effective immune response. Furthermore, the real-time imaging capabilities of MRI provide immediate feedback on treatment efficacy, which can be particularly useful in adjusting treatment parameters to optimize outcomes (Subedi et al., 2020).

The role of MRI in monitoring immune-related adverse events associated with immunotherapy is also significant. Early detection of these events can help in the timely management of side effects, improving patient safety and treatment tolerance. Studies have shown that MRI can detect cardiac and tumor-related effects of immune checkpoint inhibitors, such as myocarditis and T cell infiltration, which are crucial for assessing treatment response and differentiating between true progression and pseudoprogression (Lau et al., 2022).

### Gene therapy

Gene therapy is a medical technique that involves altering or replacing defective genes to treat or prevent tumor diseases. MRI-guided gene therapy has paved the way for precise and real-time monitoring of gene delivery, thereby optimizing surgical deployment and enhancing target coverage efficiency (Richardson et al., 2020). One significant application is tracking the distribution of viral vectors used for gene delivery, allowing for real-time assessment of how effectively these vectors reach their target tissues. Additionally, advanced MRI techniques can be employed to monitor changes in tissue structure and function following gene therapy interventions, helping to evaluate treatment outcomes (Ahrens and Bulte, 2013). Moreover, MRI’s integration has led to the development of specialized nanoparticles, including dendrimer- and copolymer-based types, aimed at improving targeting efficiency in gene delivery systems (Miranpuri et al., 2012). The combination of gene treatment mixtures, metabolic indicators, and the monitoring of objective measures guided by real-time MRI, is fast becoming a reality. This paves the way for further guiding the evolution of gene therapy (Bains et al., 2012). Furthermore, the combination of MRI-guidance and novel stereotactic aiming devices has set a strong foundation for neurological gene therapy to become an accepted practice in interventional neurology (Weber-Adrian et al., 2021). MRI has also been harnessed for gene delivery to the brain through the intravenous injection of viral vectors, enhanced by transcranial focused ultrasound to temporarily open the blood-brain barrier (Baek et al., 2020). Moreover, MRI has been employed to produce comprehensive anatomical assessments and soft tissue characterizations, offering essential insights for evaluating the effects of gene therapy on patients (Huang Y. et al., 2016). Reporter genes compatible with MRI have been used to effectively track cell delivery in therapies, assess the therapeutic impact of gene delivery, and monitor microenvironments specific to tissues or cells (Yang et al., 2016).

## Summary

Magnetic Resonance Imaging (MRI) is an essential tool in medical diagnosis, offering high-resolution images of soft tissues that facilitate the detection and evaluation of various conditions, including tumors, injuries, and neurological disorders, all without the use of ionizing radiation. A critical component of MRI technology is the use of contrast agents, which enhance image quality and accuracy by improving the visibility of internal structures and abnormalities. These agents function by altering the magnetic properties of nearby water molecules, thus increasing the contrast between different tissues. This enhanced contrast allows for better differentiation between healthy and diseased tissues, promoting more accurate diagnoses.

Traditional MRI contrast agents, particularly gadolinium-based compounds, are widely utilized; however, they often face challenges related to biocompatibility and specificity. In contrast, nanomaterial-based MRI contrast agents present several advantages, including increased sensitivity and targeted delivery. These advanced agents can be engineered with unique magnetic properties and surface characteristics, allowing for customization based on clinical needs. Moreover, nanomaterials may reduce the required dosage, thereby minimizing potential side effects while maximizing contrast enhancement. This evolution from conventional to nanomaterial-based agents signifies a promising advancement in MRI technology, paving the way for more precise diagnostics and personalized medicine.

The development of various nanomaterials—such as metal oxide nanoparticles, carbon-based nanostructures, gold nanoparticles, and quantum dots—has significantly improved the specificity and sensitivity of MRI. Each class of nanomaterials possesses distinct properties that can be leveraged for targeted imaging and therapy. This review highlights their roles in MRI-guided therapies, including targeted drug delivery systems that enhance therapeutic bioavailability at disease sites and hyperthermia treatments that utilize localized heating for improved treatment efficacy. Additionally, emerging strategies like photodynamic therapy, immunity-boosting therapies, and gene therapy showcase the potential of integrating imaging with therapeutic modalities for comprehensive patient care.

Nevertheless, each type of nanoparticle presents specific limitations that must be addressed for their effective application as MRI contrast agents. Metal oxide nanoparticles, while enhancing contrast, raise concerns regarding biocompatibility and potential toxicity due to metal accumulation in the body over time. Carbon-based materials, such as carbon nanotubes and graphene, offer excellent imaging capabilities but often struggle with functionalization, stability, and reproducibility, limiting their reliability for clinical use. Gold nanoparticles exhibit strong X-ray attenuation properties but can provoke immune responses and have limited tissue penetration, affecting their effectiveness in certain imaging scenarios. Lastly, while quantum dots possess exceptional fluorescence capabilities, their application is restricted by the risk of leaching toxic heavy metals, posing significant safety and regulatory challenges. Addressing these limitations is crucial for integrating these nanoparticles into clinical practice successfully.

Looking ahead, overcoming several challenges is vital for realizing the full potential of nanomaterial-based MRI contrast agents. Key concerns include biocompatibility, as many nanomaterials may elicit immune responses or exhibit toxicity *in vivo*, hindering their clinical applications. Additionally, the stability of nanomaterials in biological environments is a challenge, with risks of aggregation or degradation impacting performance and efficacy. Reproducibility and scalability issues in manufacturing also complicate the transition from laboratory research to clinical use. Furthermore, regulatory hurdles exist due to the unique properties of nanomaterials, necessitating thorough safety evaluations prior to medical approval. Continued research and development are essential to address these challenges and facilitate the successful integration of nanomaterials into healthcare practices.

In conclusion, nanomaterials are poised to revolutionize the field of MRI contrast agents and therapy. Their unique attributes, such as high surface area, tunable sizes, and multifunctionality, enable enhanced sensitivity and specificity in imaging techniques, allowing for earlier disease diagnosis. They can also be engineered for targeted drug delivery, reducing side effects while increasing therapeutic efficacy. Future advancements may focus on merging diagnostic and therapeutic functions within single nanomaterial platforms, leading to more personalized medicine approaches and improved patient outcomes. As research progresses, it will be imperative to tackle challenges related to biocompatibility, regulatory approval, and large-scale production to ensure the successful application of nanomaterials in clinical settings.

Overall, the future of MRI contrast agents lies in the ongoing exploration and optimization of nanomaterials, which promises to enhance both diagnostic and therapeutic outcomes in medicine. By advancing our understanding of these technologies, we stand ready to transform patient management through improved imaging strategies and targeted therapies, ultimately delivering better healthcare solutions tailored to individual patient needs.
